# Recent advances in the tandem annulation of 1,3-enynes to functionalized pyridine and pyrrole derivatives

**DOI:** 10.3762/bjoc.17.163

**Published:** 2021-09-22

**Authors:** Yi Liu, Puying Luo, Yang Fu, Tianxin Hao, Xuan Liu, Qiuping Ding, Yiyuan Peng

**Affiliations:** 1 Key Laboratory for Green Chemistry of Jiangxi Province, Key Laboratory of Functional Small Organic Molecules, Ministry of Education, Jiangxi Normal University, 99 Ziyang Road, Nanchang 330022, China; 2 Department of Gynaecology, Jiangxi Provincial People’s Hospital Affiliated to Nanchang University, 92 Aiguo Road, Nanchang, Jiangxi, 330006, China

**Keywords:** 1,3-enyne, functionalization, pyridine, pyrrole, tandem annulation

## Abstract

Great progress has been made in the tandem annulation of enynes in the past few years. This review only presents the corresponding reactions of 1,3-enyne structural motifs to provide the functionalized pyridine and pyrrole derivatives. The functionalization reactions cover iodination, bromination, trifluoromethylation, azidation, carbonylation, arylation, alkylation, selenylation, sulfenylation, amidation, esterification, and hydroxylation. We also briefly introduce the applications of the products and the reaction mechanisms for the synthesis of corresponding N-heterocycles.

## Introduction

The pyridine moiety is an important class of six-membered N-heterocycles that is widely found in many natural products, pharmaceuticals, and bioactive molecules. For instance, some pyridine derivatives have been used for therapy of HIV, cancer, inflammation, microbial infection and so on [1–5]. In addition, it is also an important synthetic unit, which is frequently used as catalyst or ligand in organic chemistry [6–10]. Therefore, the development of efficient methods for the synthesis of pyridine derivatives has attracted considerable attention [11–14]. The industrial synthetic methods of pyridines mainly involve: i) extraction from coal tar; ii) condensation of ammonia, formaldehyde, and acetaldehyde; and iii) preparation from furfural and ammonia. In addition, Hantzsch pyridine synthesis from ethyl acetoacetate, formaldehyde, and ammonia is a commonly used laboratory synthetic method. Recently, extensive and efficient methods for the construction of pyridine derivatives have been developed through the intramolecular or intermolecular tandem addition annulation/functionalization of alkynes with some N-containing compounds, such as nitriles, oximes, and imines [15–19].

The pyrrole structural motif is also an invaluable five-membered N-heterocycle that is widely used in pharmaceuticals, photoelectric materials, and functional materials [20–23]. Many pyrrole derivatives play a significant role in the life science and medicine domains due to the good bioactivities, such as antitumor, anti-HIV, and anti-HSV-1 activity [24–29]. In industry, pyrrole mainly comes from the extraction of coal tar, the condensation reaction of furan and ammonia under high temperature, or the cascade cyclization reaction of acetylene, formaldehyde, and ammonia. In the laboratory, there are many efficient methods for the synthesis of pyrrole derivatives: i) Knorr reaction: the condensation of α-aminoketones or α-amino esters in the presence of zinc powder and sodium acetate; ii) Paal–Knorr reaction: the condensation of 1,4-dicarbonyl compounds and amines, catalyzed by formic acid in anhydrous alcohol; iii) Hantzsch reaction: the condensation of α-halogenated carbonyl compounds, β-dicarbonyl compounds and amines; and iv) the latest developed multicomponent tandem reactions and transition metal-catalyzed coupling reactions [30–39].

Recently, substantial achievements have been made using azides as a powerful nitrogen source for the synthesis of various N-heterocycles, such as isoquinolines, quinolines, pyridines, pyrroles, indoles, azoles, and azepines [40–45]. 1,3-Enyne, as a powerful Michael acceptor, is a wonderful synthon for the synthesis of N-heterocycles via tandem addition and annulation. Recently, there have been several elegant reviews covering the 1,3-enynes chemistry [46–48]. For instance, Procter and co-workers reviewed the copper-catalyzed functionalization of enynes [46]. In 2020, the Wang group reviewed the development of 2-activated 1,3-enyne in enantioselective synthesis [47]. Further, the Liu group reviewed the synthesis of allenes via transition metal-catalyzed 1,4-functionalizations of unactivated 1,3-enynes [48]. In this review, we will highlight the recent advances in the tandem annulation reactions of 1,3-enyne structural motifs for the construction of functionalized pyridines and pyrroles.

## Review

### Synthesis of pyridines via tandem annulation of 1,3-enynes

**Scheme 1:**
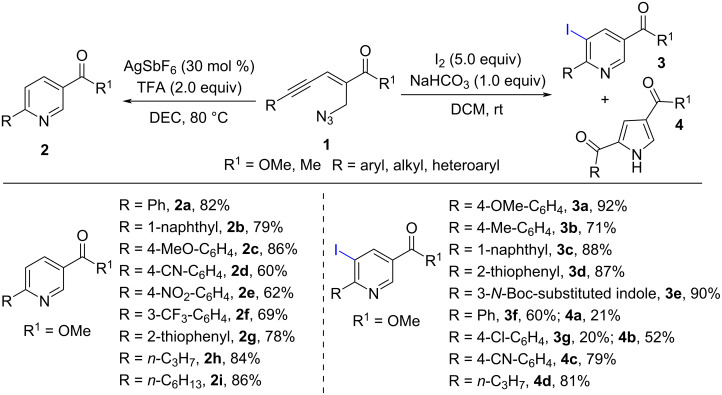
Ag/I_2_-mediated electrophilic annulation of 2-en-4-ynyl azides **1**.

In 2015, Reddy and co-workers reported the synthesis of substituted pyridines via Lewis acid-mediated aza-annulation of 2-en-4-ynyl azides **1** (Scheme 1) [49]. They discovered that Ag-mediated intramolecular annulation of 2-en-4-ynyl azides **1** could provide the corresponding 3,6-disubstituted pyridines **2** in 60–88% yield in the presence of TFA (2.0 equiv). The reaction substrates, 2-en-4-ynyl azides **1**, derived from MBH acetates of acetylenic aldehydes, could tolerate various substituted aryl, indolyl, and alkyl (such as *n*-propyl and *n*-hexyl) groups under the standard conditions. 2-En-4-ynyl azides **1** bearing electron-donating substituents (such as methyl and methoxy groups) obviously worked better than those with electron-withdrawing (such as nitro, cyano, acetyl, and trifluoromethyl) groups. Meanwhile, they also found that the aza-annulation could be carried out under iodine-mediated electrophilic annulation reaction conditions to give 5-iodo-3,6-disubstituted pyridines **3** as the major products, occasionally with a small amount of 2-acylated pyrroles **4**.

**Scheme 2:**
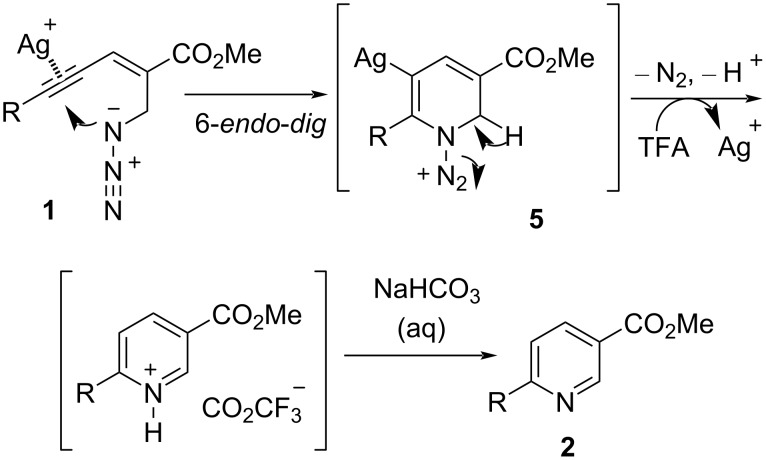
The proposed mechanism of Ag-catalyzed aza-annulation.

**Scheme 3:**
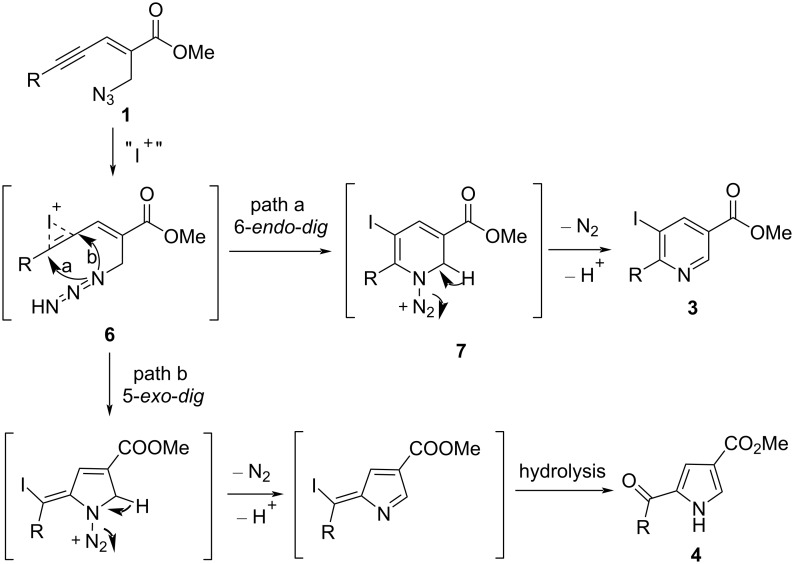
The proposed mechanism of I_2_-mediated aza-annulation.

The proposed mechanism for the Ag-catalyzed aza-annulation of (*E*)-2-en-4-ynyl azides **1** was considered to involve 6-*endo*-*dig* cyclization to give a corresponding *N*-N_2_-substituted 1,2-dihydropyridine silver salt **5**. This was protonated by TFA and the following species neutralized by base to provide a final 3,6-disubstituted pyridine product **2** (Scheme 2). However, an iodonium ion **6** was formed as a key intermediate in I_2_-mediated aza-annulations. Subsequently, the iodonium ion **6** proceeds through a 6-*endo*-*dig* cyclization to form the 5-iodopyridine **3**. On the other side, the iodonium ion **6** may undergo *5-exo-dig* cyclization to yield the 2-acylpyrrole **4**. Normally, (*E*)-2-en-4-yn-1-azides **1** with electron-rich substituent groups favorably give the 5-iodopyridine **3**, while for substrates containing electron-poor groups, the 2-acylpyrrole **4** is favored (Scheme 3).

**Scheme 4:**
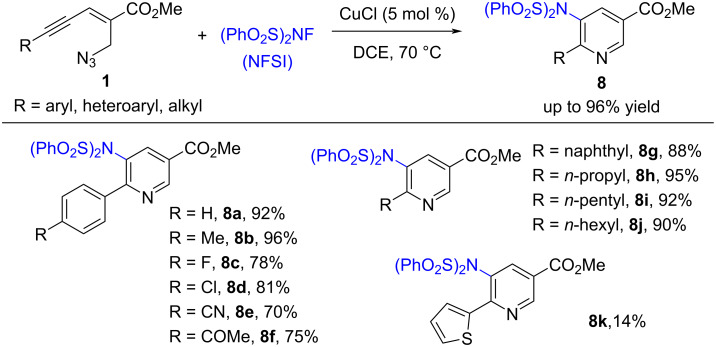
Copper-catalyzed amination of (*E*)-2-en-4-ynyl azides **1**.

Then, the Reddy group developed a copper-catalyzed aminative aza-annulation of enynyl azides with *N*-fluorobenzenesulfonimide (NFSI) to provide amino-substituted nicotinate derivatives **8** in good to excellent yield (Scheme 4) [50]. The investigation showed that the electronic effect of the residue R on the substrates influences the results significantly. (*E*)-2-en-4-ynyl azides **1** bearing electron-donating groups had better reactivity, with a higher yield and a shorter reaction time. In addition, substrates **1** with aliphatic groups (such as R = *n*-propyl, *n*-pentyl, and *n*-hexyl) were also tolerated under standard conditions, with an excellent yield.

**Scheme 5:**
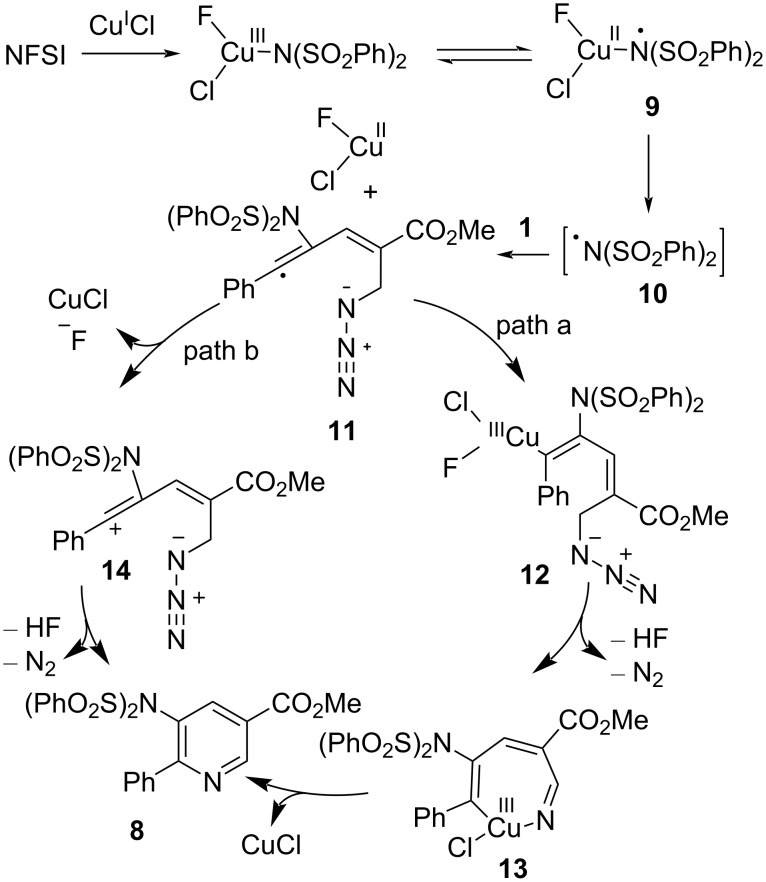
The proposed mechanism of copper-catalyzed amination.

The previous literature and control experiments showed that this aminative aza-annulation reaction may undergo a free-radical addition pathway. Firstly, NFSI oxidizes Cu(I) to form bissulfonylamidyl radical **10**. Secondly, intermolecular nitrogen free-radical addition to the alkyne provides the vinyl radical **11**. Then, there may be two possible pathways. Path a: vinyl radical **11** is trapped by Cu(II) to deliver the Cu(III) species **12**, which undergoes intramolecular annulation and reductive elimination to afford the desired product **8** and regenerate the Cu(I) catalyst. Path b: vinyl radical intermediate **11** is oxidized by Cu(II) to give the cationic vinyl species **14**. Finally, the intramolecular nucleophilic attack by azide and the following deprotonation by a fluoride anion provide the final product **8** (Scheme 5).

**Scheme 6:**
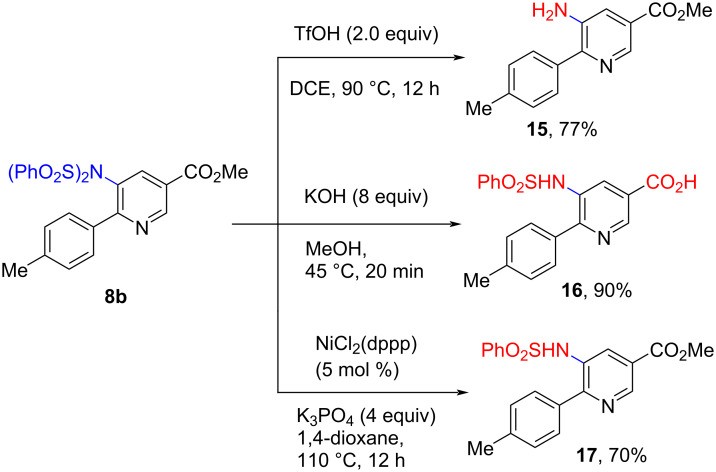
The derivatization of sulfonated aminonicotinates.

The derivatization of sulfonated aminonicotinates **8** could easily be achieved. Desulfonylation of aminonicotinate **8b** proceeded smoothly in the presence of triflic acid (2.0 equiv) in DCE at 90 °C to provide the desulfonated 5-amino-substituted nicotinate **15** in 77% yield. Furthermore, treatment of aminonicotinate **8b** with KOH (8.0 equiv) in MeOH, or with NiCl_2_(dppp) (5 mol %) and K_3_PO_4_ (4.0 equiv) in 1,4-dioxane afforded 5-(phenylsulfonamido)-6-(*p*-tolyl)nicotinic acid **16** (in 90% yield) and monodesulfonated nicotinate **17** (in 70% yield), respectively (Scheme 6).

**Scheme 7:**
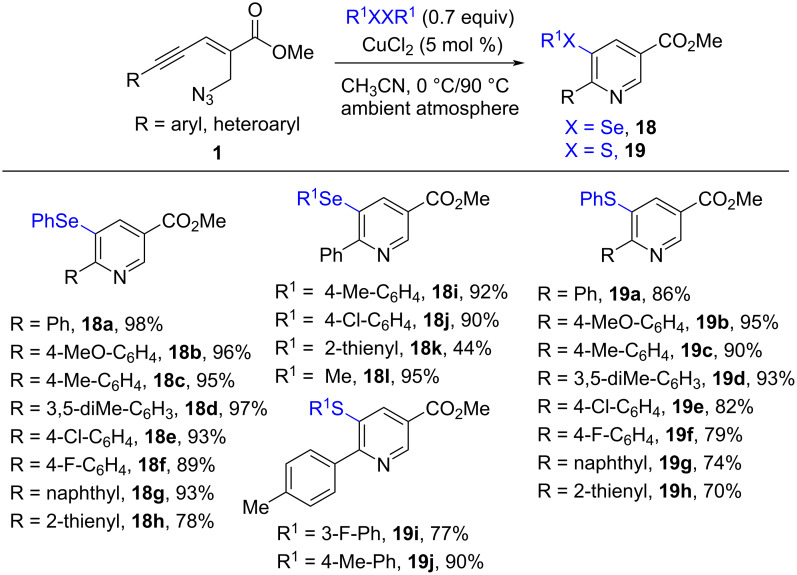
Copper-catalyzed chalcogenoamination of (*E*)-2-en-4-ynyl azides **1**.

Selenyl- and sulfenylpyridine derivatives are gaining prominence due to the prospective biological activities. They could be used for treatment of HIV, cancer, inflammation, and microbial infection. Therefore, the synthesis of selenyl- and sulfenylpyridines has attracted considerable attention. In 2019, the Reddy group reported a copper-catalyzed aza-annulation of enynyl azides **1** for the synthesis of 5-selenyl- and sulfenylpyridine derivatives **18** and **19** (Scheme 7) [51]. Diorganyl dichalcogenides (R^1^XXR^1^, X = Se, S) were used as selenyl and sulfenyl sources, respectively. The method was performed under open atmosphere to provide the target products in good to excellent yield. In comparison, the selenoamination of (*E*)-2-en-4-ynyl azides **1** showed higher reactivity and could be carried out at 0 °C in 1 h to give the selenyl-substituted nicotinates **18** in excellent yield. Electron-donating and electron-withdrawing group-substituted diaryl diselenides, 1,2-di(thiophen-2-yl)diselane, and dimethyl diselenide were compatible to give the corresponding products **18**. The sulfenylamination of (*E*)-2-en-4-ynyl azides **1** could also be carried out at 90 °C in 8 h to provide the 5-sulfenyl-substituted nicotinates **19** efficiently.

**Scheme 8:**
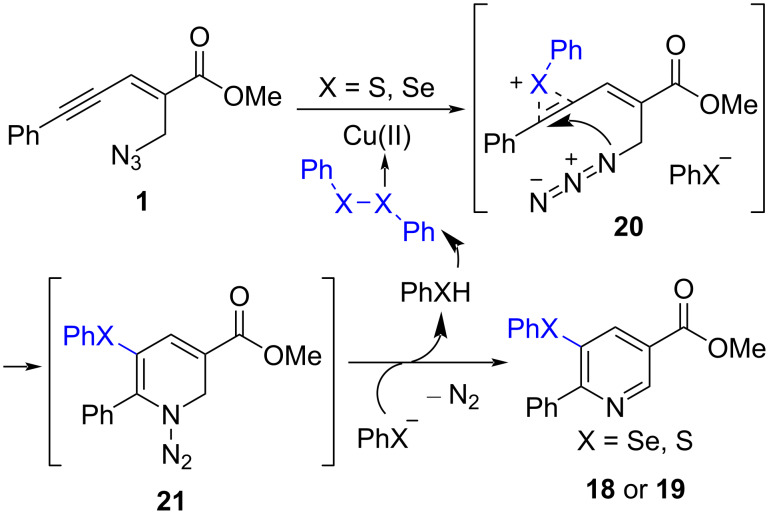
The possible mechanism of chalcogenoamination.

Based on previous literature and control experiments, the possible mechanism is outlined in Scheme 8. First, the Cu-complex-polarized X−X bond can promote the electrophilic addition onto the alkyne to generate intermediate **20**. Then, the intramolecular nucleophilic attack by azide and the following deprotonation give the final product **18** or **19**, respectively.

**Scheme 9:**
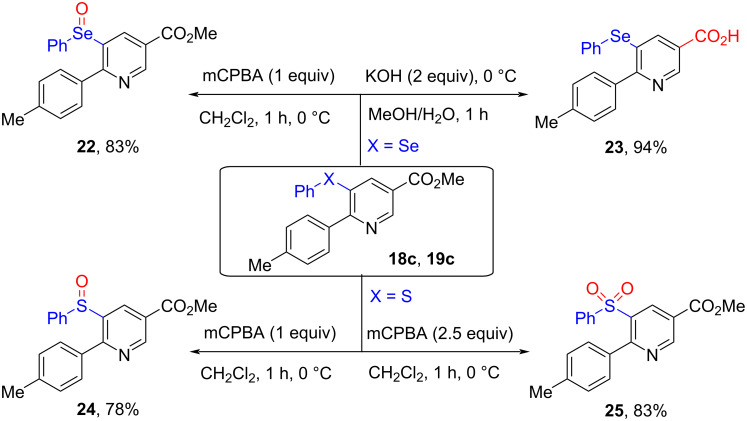
The derivatization of 5‑selenyl- and 5-sulfenyl-substituted nicotinates.

5‑Selenyl- and 5-sulfenyl-substituted nicotinates can carry out versatile transformations, which have potential application in pharmaceutical, agrochemical, and organic synthetic chemistry. For example, 5-selenyl- and 5-sulfenyl-appended nicotinates **18c** and **19c** could be oxidized by mCPBA to the corresponding selenoxide, sulfoxide, and sulfone derivatives **22**, **24**, and **25**, respectively. In addition, 5‑selenyl-substituted nicotinate **18c** could be converted to the corresponding acid **23** in 94% yield in the presence of KOH (2.0 equiv) in MeOH (Scheme 9).

**Scheme 10:**
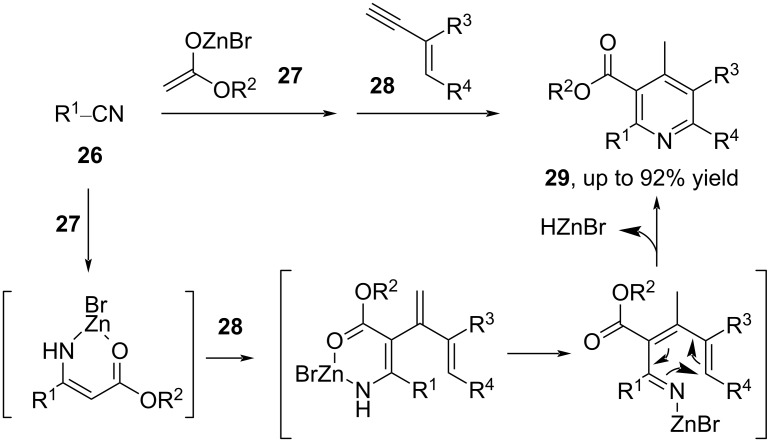
The tandem reaction of nitriles, Reformatsky reagents, and 1,3-enynes.

In 2011, Lee and co-workers reported a one-pot method for the construction of polysubstituted pyridines **29** via tandem sequential reactions of nitriles **26**, Reformatsky reagents **27**, and 1,3-enynes **28** (Scheme 10) [52]. The tandem reaction involved a regio- and chemoselective addition of the Blaise reaction intermediate to 1,3-enyne, and the following sequential processes: isomerization, cyclization, and aromatization. Both carbocyclic and acyclic 1,3-enyenes **28** were compatible to give the corresponding esterified pyridines **29** in moderate to high yield. It is worth noting that 1,3-enynes **28** bearing internal alkyne moieties were not tolerated as substrates.

**Scheme 11:**
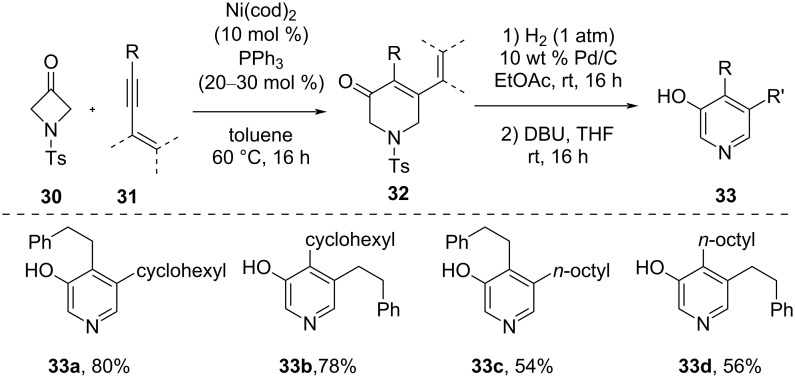
Nickel-catalyzed [4 + 2]-cycloaddition of 3-azetidinones with 1,3-enynes.

In 2016, Aïssa and co-workers reported a nickel-catalyzed [4 + 2]-cycloaddition of 3-azetidinones **30** with 1,3-enynes **31** for the synthesis of 3‑hydroxy-4,5-alkyl-substituted pyridines **33** (Scheme 11) [53]. The transformation involved a two-step sequence of successive reactions: Firstly, the nickel-catalyzed [4 + 2]-cycloaddition of 1,3-enynes **31** and *N*-Ts-substituted 3-azetidinone **30** afforded dihydropyridinones **32** in good yield. The next step involved the hydrogenation of dihydropyridinones **32** and a following desulfonylation and aromatization to give pyridine derivatives **33** in moderate to good yield.

### Synthesis of pyrroles via tandem annulation of 1,3-enynes

**Scheme 12:**
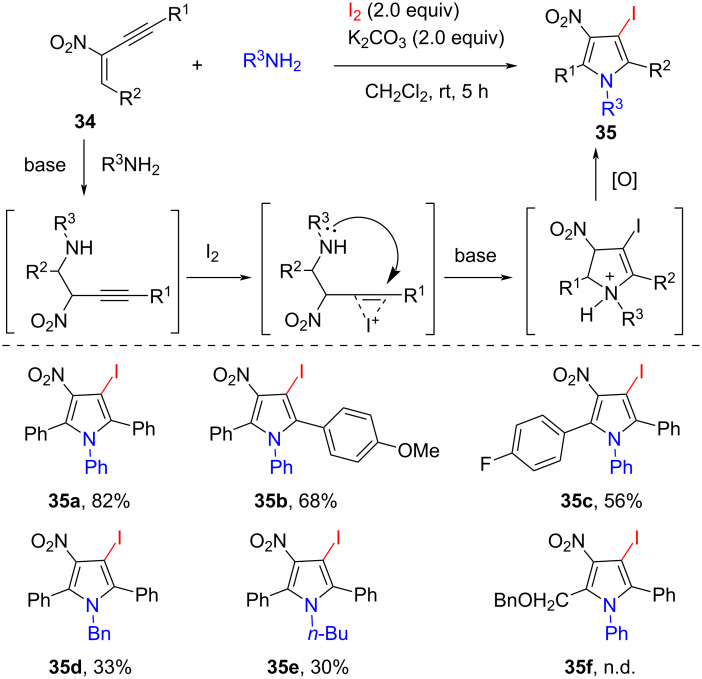
Electrophilic iodocyclization of 2-nitro-1,3-enynes to pyrroles.

Recently, great achievements have been made in electrophilic iodocyclization of alkynes for the synthesis of five- or six-membered cyclic compounds [54]. Various efficient synthetic methods have been developed for the synthesis of halogenated pyrroles, which are widely presented in many pharmacologically active natural products, bioactive molecules, and organic building blocks. Based on the previous studies on heterocycle synthesis, Punniyamurthy and co-workers designed the electrophilic iodocyclization of 2-nitro-1,3-enynes **34** for the synthesis of pyrrole derivatives. In 2013, they reported an efficient route to pentasubstituted pyrroles from 2-nitro-1,3-enynes **34**, amines, and iodine under mild conditions (Scheme 12) [55]. The reaction was performed in CH_2_Cl_2_ at ambient conditions in the presence of 2.0 equiv of K_2_CO_3_. Substrates bearing electron-donating or electron-withdrawing groups were compatible under standard conditions to give the highly substituted pyrroles in moderate to good yield. Aliphatic amines were also tolerated, providing the desired products in only moderate yield. The plausible mechanism involves a tandem base-promoted aza-Michael addition, 1,2-iodocyclization, and iodine-mediated oxidative aromatization.

**Scheme 13:**
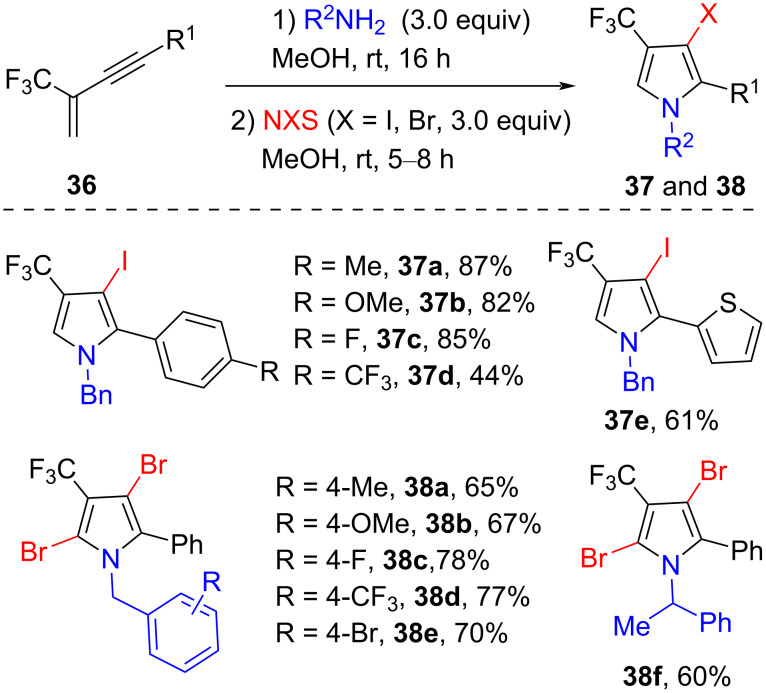
Electrophilic halogenation of 2-trifluoromethyl-1,3-enynes to pyrroles.

In 2017, Zhang and co-workers reported a silver-catalyzed tandem reaction of 2-trifluoromethyl-1,3-enynes **36** with primary amines, affording various trifluoromethyl-substituted 3-pyrrolines [56]. Subsequently, they also developed a novel route for the synthesis of halogenated trifluoromethylated pyrroles **37** and **38** by sequential intermolecular hydroamination reaction of 2-trifluoromethyl-1,3-enynes **36** with aliphatic primary amines and the following NXS-mediated oxidative cyclization (Scheme 13) [57]. The method tolerated various substituted benzylamines, 2-phenylethanamines, isopropylamine, and other aliphatic chain-like amines. Furthermore, both furan-2-ylmethanamine and thiophen-2-ylmethanamine were reacted smoothly with NIS under standard conditions, while they did not react well with NBS. Notably, under the same reaction conditions, the desired products of the iodination and bromoniation reactions were trifluoromethylated monoiodopyrroles **37** and dibromopyrroles **38**, respectively.

**Scheme 14:**
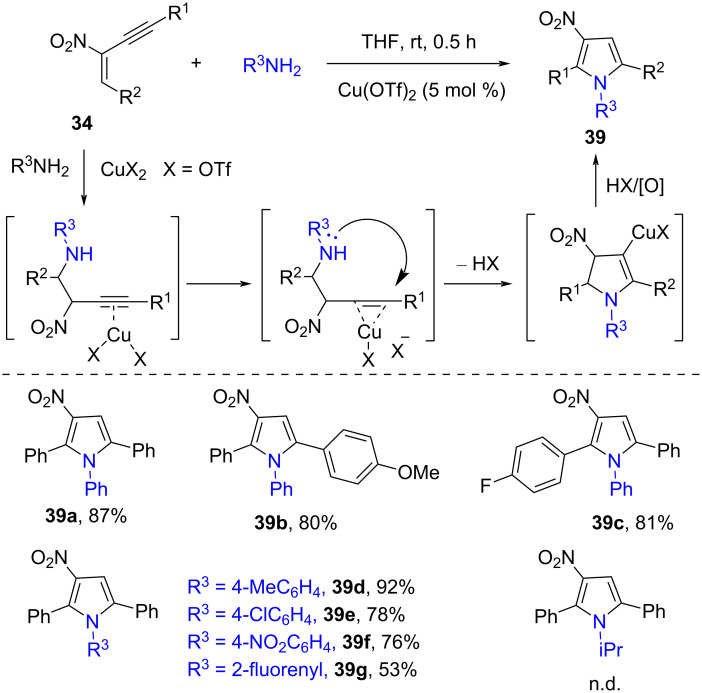
Copper-catalyzed cascade cyclization of 2-nitro-1,3-enynes with amines.

Subsequently, Punniyamurthy and co-workers also described the copper-catalyzed cascade cyclization of 2-nitro-1,3-enynes **34** to tetrasubstituted pyrroles **39** (Scheme 14) [58]. Through screening the conditions, the Cu(OTf)_2_-promoted (5 mol %) annulation addition reaction of 2-nitro-1,3-enynes **34** and amines was carried out smoothly in THF at room temperature under air. The protocol showed broad substrate scope, and various different aromatic substrates (R^1^, R^2^, and R^3^ = aryl) reacted well. However, no target product was observed when aliphatic amine was used as substrate under standard conditions. The proposed catalytic cycle included aza-Michael addition of arylamines, Lewis acid copper(II)-catalyzed intramolecular 5-*endo*-*dig* cyclization, protonation, and oxidation to provide the final products, tetrasubstituted pyrroles **39**.

**Scheme 15:**
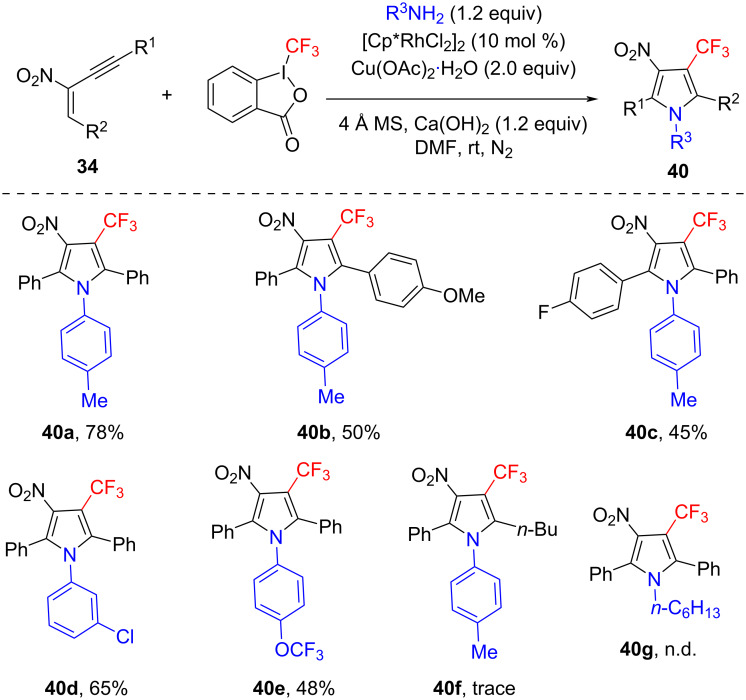
Tandem cyclization of 2-nitro-1,3-enynes, Togni reagent II, and amines.

The introduction of a trifluoromethyl group into organic molecules can efficiently modify the physical, chemical, and biological properties of the compounds. A trifluoromethyl-substituted pyrrole unit is widely present in many natural compounds and pharmaceuticals with high biological activity. Based on our previous study on the construction of trifluoromethylated coumarins [59], we recently developed a Rh-catalyzed approach to trifluoromethyl-substituted pyrroles using the Togni reagent II as trifluoromethyl source. It involves a three-component cascade reaction of 1,3-enynes, anilines, and Togni reagent II to afford fully substituted pyrrole derivatives in DMF at room temperature (Scheme 15) [60]. The reaction was promoted by Cu(OAc)_2_·H_2_O (2.0 equiv) and Ca(OH)_2_ (2.0 equiv), providing the desired products in moderate to good yield. Various substituted 1,3-enynes with methyl, methoxy, fluoro, and chloro groups could react with *p*-toluidine under standard conditions in moderate yield (40–71%). Notably, aromatic amines bearing electron-donating or electron-withdrawing groups were compatible. When 2-naphthylamine was used under standard conditions, α-trifluoromethyl-substituted 2-naphthylamine was obtained as major product (51% yield), and only a trace amount of the desired product was observed. Control experiments indicated that the possible reaction mechanism may proceed through a Cu(II)/Rh(III)-promoted radical process.

**Scheme 16:**
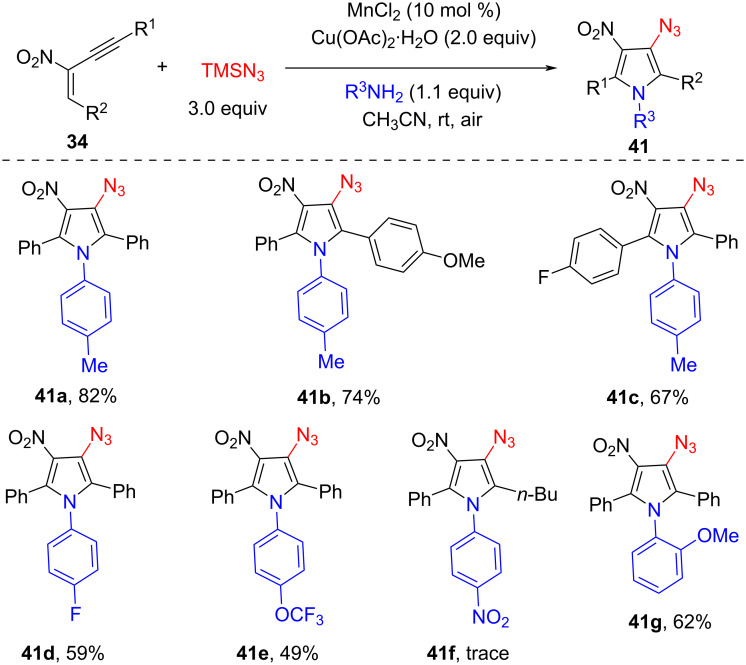
Tandem cyclization of 2-nitro-1,3-enynes, TMSN_3_, and amines.

Aryl azides are versatile intermediates, which were widely used in synthetic and medicinal chemistry as well as material and biological sciences. Recently, great attention has been paid to synthesize organic azido compounds via various transformations. In 2020, we reported an efficient method for the synthesis of fully substituted azidopyrroles **41** via Cu- and Mn-co-mediated aerobic oxidative cyclization/azidation reaction of 2-nitro-1,3-enynes **34** with amines, and trimethylsilyl azide (TMSN_3_, Scheme 16) [61]. The reaction could be carried out efficiently in the presence of 2.0 equiv of Cu(OAc)_2_·H_2_O. Interestingly, the addition of 10 mol % MnCl_2_ could promote the reaction more smoothly. A wide range of substituted aromatic amines were reacted well, while amines substituted with strongly electron-withdrawing (such as nitro and trifluoromethyl) groups, heteroaryl amines, and aliphatic amines were not compatible. Control experiments showed that the addition of 2.0 equiv of (2,2,6,6-tetramethylpiperidin-1-yl)oxyl (TEMPO) under standard conditions could inhibit the formation of target product. In contrast, the compound TEMPO–N_3_ was detected by GC–MS analysis. Based on the radical trapping experiment and previous reports, the reaction may undergo a radical process.

**Scheme 17:**
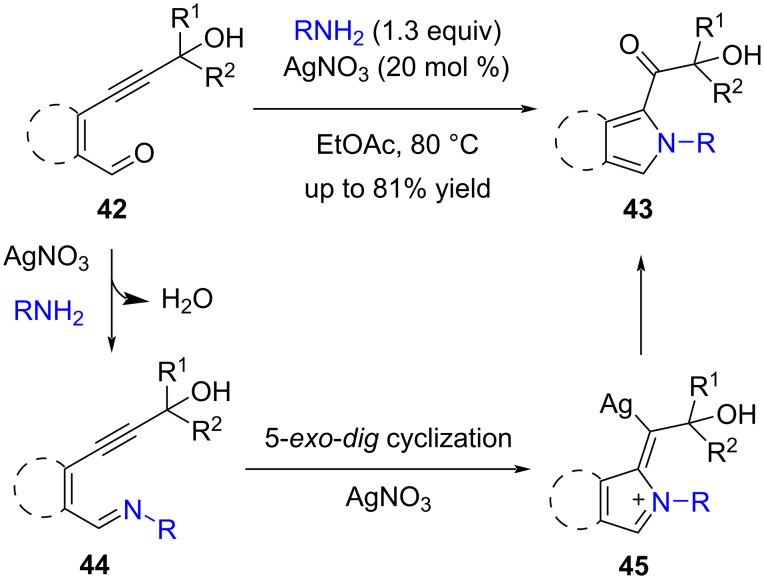
Cascade cyclization of 6-hydroxyhex-2-en-4-ynals to pyrroles.

2-Carbonylpyrrole is a key subunit of many bioactive natural products with potential biological activities or pharmacological activities. For instance, longanlactone, zomepirac (Zomax), ketorolac and pollenopyrroside A are pyrrole derivatives bearing a 2-carbonyl group. Therefore, the synthesis of such kinds of pyrrole derivatives is highly valuable. In 2017, Baire and Gandhi reported an Ag-catalyzed cascade cyclization of 6-hydroxyhex-2-en-4-ynals **42** and primary amines to give the 2-(α-hydroxyacyl)pyrroles **43** in moderate to good yield (Scheme 17) [62]. The proposed mechanism involves the condensation of amine and aldehyde to give the imine **44** and the AgNO_3_-promoted 5-*exo*-*dig* cyclization of imine to form a zwitter ion intermediate **45**.

**Scheme 18:**
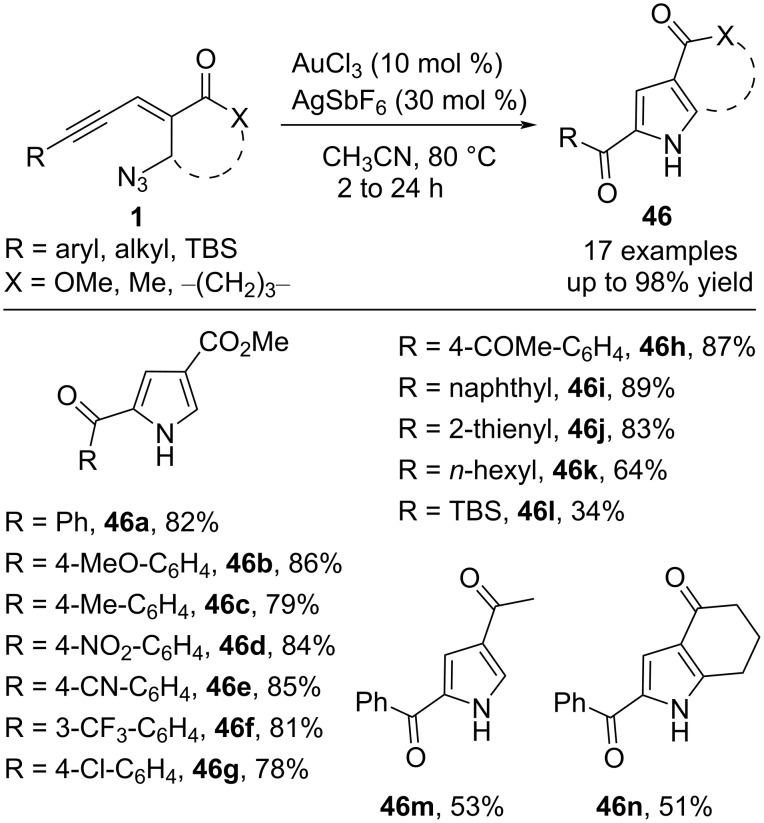
Au/Ag-catalyzed oxidative aza-annulation of 1,3-enynyl azides.

In 2017, the Reddy group also reported a method for the construction of 2-carbonylpyrroles **46** through Au/Ag-catalyzed intramolecular oxidative aza-annulation of 1,3-enynyl azides **1** (Scheme 18) [63]. The method is very applicable, and various aryl-substituted enynyl azides bearing electron-donating or -withdrawing (such as methyl, methoxy, chloro, cyano, nitro, acyl, and trifluoromethyl) groups all worked smoothly to deliver the corresponding 2-carbonylpyrroles **46** in good to excellent yield. Aliphatic enynyl azide (R = 1-hexyl) was also tolerated efficiently under the standard conditions to afford the desired product **46k** in 64% yield. In addition, *tert-*butyldimethylsilyl (TBS)-substituted enynyl azide provided the target product **46l** in 34% yield.

**Scheme 19:**
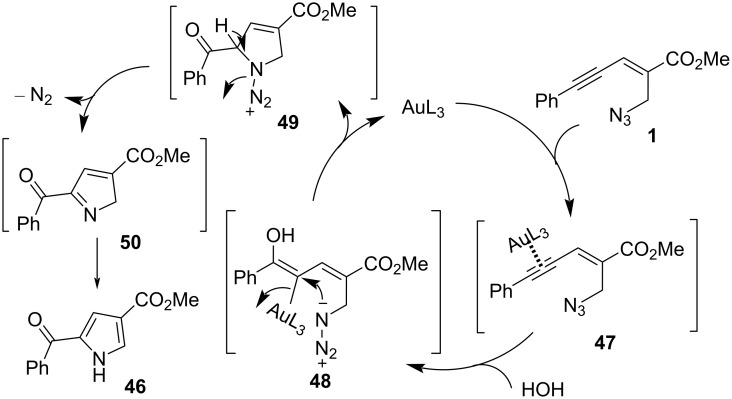
The plausible mechanism of Au/Ag-catalyzed oxidative aza-annulation.

The transformation involves a sequence of C−N/C−O bond formation, and the corresponding plausible mechanism is shown in Scheme 19. Firstly, Au-coordinated alkyne undergoes regioselective hydration to form intermediate **48**. Then, intramolecular nucleophilic attack by azide occurs to give 2-carbonyl intermediate **49**. Subsequently, intermediate **49** will undergo aromatization as well as the release of a nitrogen molecule to form the desired product **46**.

**Scheme 20:**
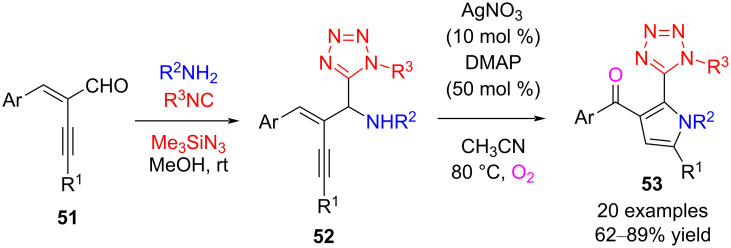
Synthesis of 2-tetrazolyl-substituted 3-acylpyrroles from enynals.

In 2018, Ding and co-workers reported the synthesis of 2-tetrazolyl-substituted 3-acylpyrroles **53** via sequential Ugi-azide/Ag-catalyzed oxidative cycloisomerization reactions in good yield (Scheme 20) [64]. Firstly, The Ugi-azide reaction products **52** were obtained efficiently through the cascade reactions of enynals **51**, primary amines, aliphatic isocyanides, and trimethylsilyl azide. The following reaction involves Ag-catalyzed intramolecular 5-*endo*-*dig* cyclization and base (DMAP)-promoted oxidative isomerization. The presence of DMAP is necessary for this transformation.

**Scheme 21:**
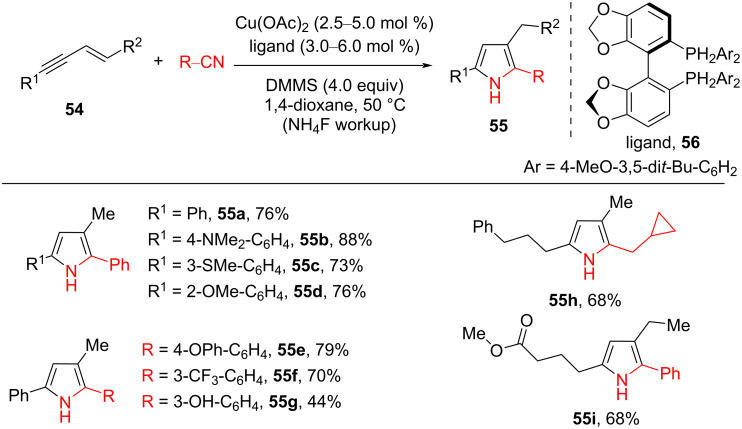
CuH-catalyzed coupling reaction of 1,3-enynes and nitriles to pyrroles.

Recently, copper hydride (CuH) catalysis has been a wonderful procedure for olefin hydrofunctionalization via the formation of nucleophilic alkylcopper intermediate. In 2016, Buchwald and co-workers described a CuH-catalyzed asymmetric addition of olefin to ketones [65]. Then, they also reported another CuH-catalyzed coupling reaction of 1,3-enynes **54** and nitrile to prepare polysubstituted pyrroles **55** (Scheme 21) [66]. The substrates **54** could be easily prepared by Sonogashira coupling of terminal alkynes and vinyl halides. It is worth mentioning that the addition of the bisphosphine ligand DTBM-SEGPHOS (**56**) was very important to promote the transformation efficiently. The reaction showed a broad substrate scope, with aromatic and aliphatic substrates **54** (R^1^ = aryl, heterocycle, and alkyl) being good coupling partners, providing the corresponding 2,3-dialkyl-5-aryl-substituted pyrroles **55** in moderate to good yield. In addition, the method could tolerate a wide range of functional groups, such as phenolic hydroxy, aryl bromide, ester, terminal olefin, aryl chloride, and silyl-protected alcohol moieties. Furthermore, both aromatic and aliphatic nitriles performed well in the reaction with 1,3-enynes **54**, providing moderate yield and regioselectivity. The CuH-catalyzed intramolecular coupling of enyne containing a nitrile group worked smoothly and gave a moderate yield under standard conditions at a decreased concentration.

**Scheme 22:**
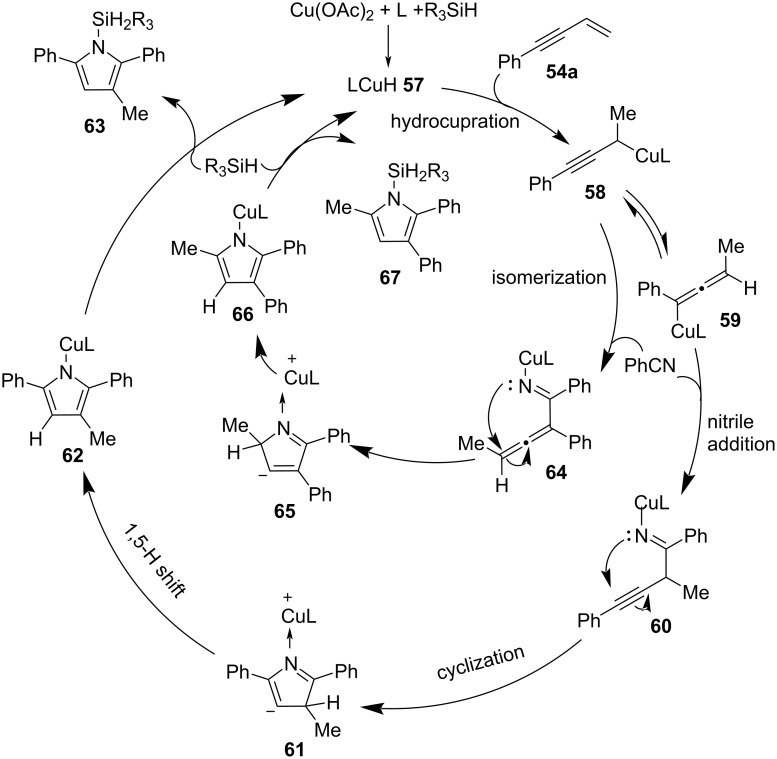
The mechanism of CuH-catalyzed coupling of 1,3-enynes and nitriles to pyrroles.

A plausible mechanism according to previously reported methods is proposed in Scheme 22. Firstly, the hydrocupration of enyne **54** with LCuH **57** provides propargylcopper intermediate **58**. The 1,3-isomerization of **58** and the following nitrile addition produces imine intermediate **60**, which subsequently undergoes intramolecular cyclization, a 1,5-hydrogen shift and σ-bond metathesis with hydrosilane to give the silylated pyrrole product **63** and the LCuH catalyst **57**. In addition, the intermediate **58** might go through isomerization to form imine intermediate **64**, which undergoes intramolecular cyclization to provide the minor regioisomer **67** (inner cycle in Scheme 22).

## Conclusion

1,3-Enynes, one of the most significant classes of Michael acceptors for the construction of N-heterocycles, have been widely used in organic synthesis. We herein reviewed the recent advances in the development of tandem cyclization reactions of 1,3-enynes in the presence of electrophiles or Lewis acid catalysts to form pyridines and pyrroles. Series of iodinated, aminated, selenylated, sulfenylated, esterified, and hydroxylated pyridine derivatives have been prepared based on 1,3-enynes. In addition, we also reviewed the tandem cyclization of 1,3-enynes to realize various functionalizations of pyrrole derivatives, such as iodination, bromination, trifluoromethylation, azidation, carbonylation, arylation, and alkylation. The proposed mechanism generally involves two kinds of intramolecular cyclizations: one is 6-*endo*-*dig* cyclization to promote the formation of pyridine ring derivatives and the other is 5-*exo*-*dig* cyclization to afford the pyrrole derivatives.

Considering the good biological activities and the wide applicability in synthetic organic chemistry, biopharmaceuticals, and materials, it is desirable to develop more efficient methods for the synthesis of diverse functionalized pyridine and pyrrole derivatives from easily available 1,3-enynes. Therefore, the significant challenges will focus on the following aspects in the future: i) development of more functionalizations of pyridines and pyrroles (such as fluorination, trifluoromethylthiolation, olefination, alkynylation, boronization, phosphorization, etc); ii) discovery of new transformations of 1,3-enynes to other N-heterocycles; and iii) more extensive investigations into the mechanism.
